# Q&A: Auxin: the plant molecule that influences almost anything

**DOI:** 10.1186/s12915-016-0291-0

**Published:** 2016-08-10

**Authors:** Sebastien Paque, Dolf Weijers

**Affiliations:** Laboratory of Biochemistry, Wageningen University, Stippeneng 4, 6708 WE Wageningen, The Netherlands

## Abstract

Auxin is an essential molecule that controls almost every aspect of plant development. Although the core signaling components that control auxin response are well characterized, the precise mechanisms enabling specific responses are not yet fully understood. Considering the significance of auxin in plant growth and its potential applications, deciphering further aspects of its biology is an important and exciting challenge.

## What is auxin?

A conservative definition is that auxin is indole acetic acid (abbreviated IAA), a weak organic acid with a structure similar to the amino acid tryptophan. It possesses an indole ring and a carboxylic acid function. A broader definition of auxin(s) would be a class of compounds that impact plant development in the way IAA does. Other natural auxins, such as 4-Cl-IAA and PAA, have been identified in plants, and several synthetic compounds such as NAA or 2,4-D have IAA-like activity and are widely used in horticulture, agriculture, and research.

IAA, the most studied auxin, is extremely potent in controlling many aspects of plant growth and development, despite its relatively simple chemical structure. It controls cell division, cell expansion, and cell differentiation. It has a ubiquitous and context-dependent function, making it difficult to assign a single function to auxin.

## How was auxin discovered?

IAA was isolated from maize by chemists during the 1930s, but its existence had been hypothesized several decades earlier. For example, Charles and Francis Darwin hypothesized the existence of a mobile signal that promotes elongation of grass coleoptiles. In simple and elegant experiments, father and son showed that coleoptiles bend to the light source when illuminated from one direction. Other scientists, including Boyen-Jensen, Paal, and Went, independently used the same experimental system to show that the bending was promoted by a mobile signal that was hydrophilic in nature, and this signal was finally identified as IAA [1].

These early discoveries have spurred the development of a lively and active research field that has made remarkable discoveries in the past decades.

It appears that auxin affects almost all developmental steps in plants from early embryogenesis to fruit ripening and controls organogenesis at the meristems, which define plant architecture. Nowadays, research focuses on understanding how such a small molecule can be ubiquitous and at the same time have context-dependent function.

## Are auxin levels the same in all plant cells?

No, you do not find the same amount of auxin in all the tissues of a plant. In fact, the uneven auxin distribution is a key factor for proper development. IAA concentrations can differ by an order of magnitude between shoot and root and appear highest in meristems located at the tip of the roots and the shoots. Even though many cell types seem able to produce auxin [2], the capacity in young leaves is comparatively high. This freshly made auxin is then transported from source organs (such as young leaves) to sink organs (such as meristems) where auxin accumulates. In those organs, levels of auxin differ between cell types [3]. This heterogeneity in auxin levels is due to directional auxin transport mediated by specific families of influx (Aux/LAX proteins) or efflux (PIN proteins) regulators located on polar domains in the cell membrane [4]. The precise positioning of these auxin channels orient auxin flux and create heterogeneity for IAA distribution.

High auxin levels are especially present in the center of the root meristem, also known as the quiescent center where stem cells are embedded. From this zone auxin concentration tends to decrease. Hence, a gradient forms that is thought to be crucial for establishing a proper developmental pattern and maintain stem cell niches.

**Fig. 1:**
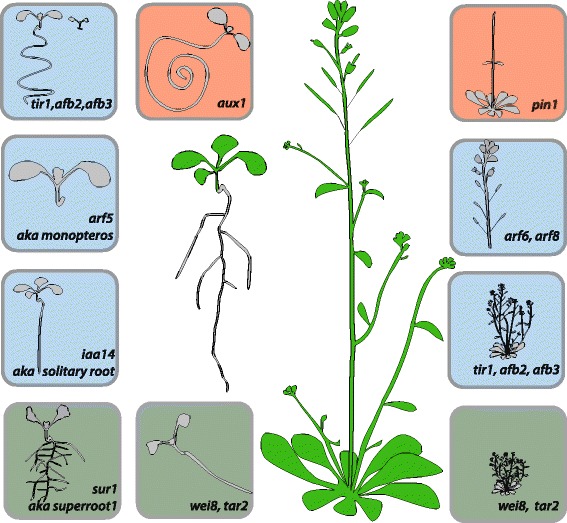
Auxin homeostasis is essential for proper development. Drawings of a wild-type seedling and an adult plant (*center* of the figure in *green*) and some characterized mutants for auxin homeostasis (in *boxes*). Mutants with affected auxin biosynthesis are in *light brown boxes. sur1* is an overproducer [5] while *wei8 tar2* [6] is depleted of auxin. Signaling mutants are shown in *light blue boxes* and are mutated in the auxin transcription factor 5 (*arf5/monopteros*) [25], auxin transcriptional inhibitor *iaa14* (or *solitary root*) [26], or the auxin co-receptors (*tir1*, *afb2*, *afb3*) [27]. Mutants for influx (*aux1*) [28] and efflux auxin transporters (*pin1*) [7] are shown in the *light red boxes*

Homeostasis of auxin is also regulated by auxin conjugation. IAA is then coupled to an amino acid and stored or degraded. Any modification of its homeostasis will lead to dramatic phenotypic changes, as exemplified by mutants that overproduce auxin (e.g. *superroot1* [5]), that are depleted in auxin (e.g. *wei8 tar2* [6]), or in which auxin transport is disturbed (e.g. *pin1* [7]) (Fig. 1). These phenotypes highlight the importance of controling auxin distribution in plant development. However, how the cell senses IAA and responds to auxin stimulus are also critical questions.

## How do plant cells sense auxin?

The central role of auxin in plant development makes the quest towards understanding the mechanisms underlying its action a fascinating and challenging one. Many studies in the past decades have led to a comprehensive insight into how auxin is perceived and how cells respond to auxin. It is well established that auxin can trigger very fast non-transcriptional responses, such as activation of the plasma membrane proton pump and ion channels, as well as the reorientation of microtubules [8]. On the other hand, it has become clear that many of the developmental responses to auxin are mediated by changes in the expression of thousands of genes [9]. These non-transcriptional and transcriptional responses may be interconnected and a major future challenge will be to define how cells merge these two pathways.

Since the late 80s and the use of genetics in the model plant *Arabidopsis*, impressive progress has been made in the understanding of transcriptional auxin signaling and many components have been identified. Surprisingly, it appears that only three dedicated molecular components are required to reconstruct a minimal nuclear auxin pathway (NAP) in yeast [10].

**Fig. 2:**
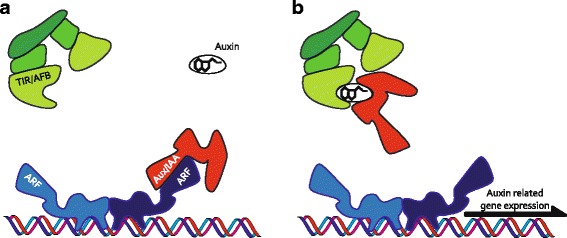
The nuclear auxin pathway (NAP) is the machinery that controls auxin gene expression. Without auxin (**a**), the transcriptional functions of the auxin response factors (*ARF*, in *blue*) are inhibited by the Aux/IAA proteins (in *red*) through heterodimerization. When auxin enters the nucleus (**b**), it allows binding between Aux/IAAs and the SCF (TIR/AFBs) complex (in *green*). These co-receptors will subsequently be degraded, allowing the ARF to modulate auxin-related gene expression

The first of these are the DNA-binding auxin response factors (ARFs), which are in charge of regulating auxin-dependent genes. When auxin levels are low in the nucleus, Aux/IAA proteins inhibit ARF activity by heterodimerizing with the ARF and blocking auxin-dependent gene expression. Auxin unlocks the ARFs by promoting binding between the Aux/IAA and TIR1/AFB proteins that together form the auxin co-receptor complex. TIR/AFB proteins are substrate-binding subunits of E3 ubiquitin ligases. The formation of this complex will trigger poly-ubiquitination of Aux/IAA, followed by its degradation. This simple system seems to account for much of the regulation in auxin-dependent gene expression (Fig. 2).

However, other potential signaling components, such as IBR5 and ABP1, have been characterized in the past. These do not seem to be part of the NAP and their precise role in auxin signaling is still a matter of debate [11].

## How can such a simple pathway control a large number of developmental processes?

This question is perhaps the most intriguing and pressing one in current auxin research. Several new studies have provided insights that help address this question. Even if the NAP appears rather simple, it is in fact highly feedback-regulated and encapsulates much diversity, such that a complex array of outputs can be generated [9].

One level for diversification in response is that the three central actors in NAP belong to multigene families, with six TIR/AFB, 29 Aux/IAA, and 23 ARF proteins in *Arabidopsis*, and also that different gene expression patterns give each tissue a specific set of NAP components [12]. Besides this differential expression, biochemical studies, as well as reconstruction experiments in a synthetic yeast system, also demonstrated that these different actors of the NAP have different intrinsic properties, for example, in interaction affinities among the three core components [9].

These specific interaction capacities bring an incredible complexity to the NAP and seem to be a cornerstone that gives dynamic range and specificity to the system. A key element of specificity is found in the ARF transcription factors, which are the output of the NAP. They are not interchangeable and mutations lead to distinct phenotypes, indicating that they are important factors in determining pathway specificity [13]. One explanation could lie in the DNA binding properties of the ARFs, which appear to be different among the ARF family. Indeed, the set of DNA binding motifs identified for ARFs has recently been expanded using genome-wide analysis and in vitro approaches [14–16]. Even though ARF proteins have similar intrinsic DNA binding specificities, it recently became apparent that differences in DNA binding properties may arise from the ARFs binding DNA as dimers with distinct preference for spacing between two adjacent binding sites [14, 15].

These recent breakthroughs in the field suggest new explanations for how such a simple-in-appearance pathway could control such a wide variety of developmental processes and give rise to diversity in auxin response.

## Is auxin specific to plants?

While auxin is a key regulator of plant development, IAA and genes involved in its biosynthesis are also found in a wide range of different bacteria or fungi. While auxin can impact gene expression in some bacteria, it does not seem to be used as a growth signal there, but rather as a signal to communicate with plants in an ecological context [17]. Furthermore, IAA biosynthesis is used by some pathogenic bacteria to hijack plant development (for example, the crown galls induced by *Agrobacterium tumefaciens*) in a range of plant species. Thus, the question becomes whether all plant species respond to auxin. There is no clear answer to this question yet, even though the presence of auxin and auxin response have been reported in algae [18]. Without genomic, genetic, and biochemical investigations, it is not possible to tell if such responses are based on conserved mechanisms.

However, auxin response is clearly ubiquitous in all flowering plant species investigated, with *Arabidopsis*, maize, and rice being focal species. Recently, it has been found that a very similar auxin response pathway operates in the earliest diverging land plants, the liverworts and mosses. Rather strikingly, while the moss *Physcomitrella patens* still has some degree of genomic complexity in its NAP [19], the liverwort *Marchantia polymorpha* appears to have a nearly minimal set of NAP components [20].

Thus, auxin response has an ancient history but critical questions remain unanswered as to the origin of auxin response and how different sets of genes have become auxin-dependent during plant evolution. So far, only orthologs of auxin signaling that are not part of the NAP have been found in algae [18] and nothing is known about the emergence and evolution of the NAP components. Hopefully, the steady release of genome or transcriptome [21] sequences thanks to new sequencing technologies will help to answer these questions.

## Is auxin a hormone, a morphogen, or something else?

Many signaling molecules have been identified in the animal kingdom, as well as in prokaryotes. In animals, several such molecules are referred to as “hormones”, some as “morphogens”. These terms are often used in the literature to describe auxin, but it is fair to ask whether they are suitable. It is important to note that both hormone and morphogen concepts have very specific connotations in the animal kingdom.

In simple terms, a hormone is usually produced in a specific tissue, often transported (e.g., via the circulatory system), should lead to precise output in distant cells, and be capable of acting at low concentrations.

Auxin’s characteristics don’t exactly fit within a strict hormone definition. Although auxin may act at low concentrations and can be transported, it is not produced in a specific tissue. Auxin may also be too pleiotropic to be considered a hormone. Indeed, the number of genes affected by auxin is high and varied, depending on the tissue, and the developmental processes in which it is involved are diverse, as are the ways auxin affects these processes. Thus, it is not possible to attribute a specific function to auxin. In fact, auxin rather appears to be a signal that triggers a pre-set system than a hormone with a specific function [22].

The same issues arise when considering auxin as a morphogen. A morphogen is a compound that can modify cell specification (pattern formation) in a concentration-dependent manner, with different concentrations leading to different outputs. As such, cells can “read” their position using the morphogen gradient and use this information to define their state or identity.

Auxin response can be found in what could be considered gradients [3, 23] and this has led to speculation that auxin may act in a concentration-dependent manner to instruct cells along those gradients. While attractive, the evidence supporting a morphogen-like action is limited. If auxin concentration alone can instruct cells, then the capacity to respond should be uniform. This is clearly not the case, as all components of the NAP vary in their expression patterns and can, therefore, confer distinct downstream effects on cells. Furthermore, while dose-dependent action is conceivable, it has not yet been shown that cells can respond in unique ways to specific concentrations. Finally, the auxin pathway is subject to intense feedback regulation or cross-talk with other signaling pathways, which makes linking concentrations to output problematic.

To conclude, the precise definition of auxin is still complicated due to its pleiotropic effects. Considering its crucial role in plant development and the difference between plants and animals, perhaps auxin deserves a proper adjective not related to the animal field, such as the often-used term “phytohormone”. Or maybe we should not care about the terminology and appreciate the molecule for the plant growth substance it is.

## Why is it so important to study auxin?

Because of its potent impact on cell division, cell growth, and differentiation, auxin is very commonly used for artificially controlling plant growth. The most common use of auxin in our daily life is in growing plants from cuttings. Gardeners often use a powder to stimulate root proliferation; this is essentially auxin at low concentration. As ever, the dose makes the poison and, at high concentrations, synthetic auxins like 2,4-D are used as herbicides to which dicotyledonous plants are much more sensitive than monocotyledonous plants. An infamous example is “Agent Orange” used for defoliation during the Vietnam War.

As auxin induces cell division at physiological concentrations, it can be used in a balanced cocktail with another growth regulator, cytokinin, to promote cell proliferation in cell culture or in vitro propagation. This knowledge allowed, for instance, the emergence of low cost orchids and virus-free potatoes.

Detailed knowledge of the mechanism of action of auxin has recently also led to powerful new technology in non-plant laboratories. The ability of IAA to rapidly and efficiently induce the degradation of Aux/IAA proteins without the requirement of plant-specific components other than TIR1 led animal and yeast biologists to import this system to rapidly and conditionally target the degradation of other proteins when coupled to a small domain of an Aux/IAA protein [24].

Finally, considering the key role of auxin in plant development, understanding how auxin works will help in elucidating how fundamental developmental processes are controlled. Besides the fascination in revealing how a complex living organism is wired, one can conceive ways to engineer plant development using knowledge gained from auxin-dependent processes. But this is one idea among many that this fascinating molecule may lead us towards in the future.
